# Transcatheter Mitral Valve Repair Simulator Equipped with Eye Tracking Based Performance Assessment Capabilities: A Pilot Study

**DOI:** 10.1007/s13239-021-00549-4

**Published:** 2021-06-07

**Authors:** Jan Michael Zimmermann, Mattia Arduini, Luca Vicentini, Francesco Maisano, Mirko Meboldt

**Affiliations:** 1 Product Development Group Zurich, Department of Mechanical and Process Engineering, ETH Zurich, Tannenstrasse 3, CLA G17.2, 8092 Zurich, Switzerland; 2 Cardiac Surgery, University of Zurich, Zurich, Switzerland

**Keywords:** Simulation training, Education, Objective assessment, Mitral valve, MitraClip, Transseptal puncture

## Abstract

**Background:**

The increase in cardiovascular disease cases that require minimally invasive treatment is inducing a new need to train physicians to perform them safely and effectively. Nevertheless, adaptation to simulation-based training has been slow, especially for complex procedures.

**Objectives:**

We describe a newly developed mitral valve repair (MVR) simulator, equipped with new objective performance assessment methods, with an emphasis on its use for training the MitraClip™ procedure.

**Methods:**

The MVR contains phantoms of all anatomical structures encountered during mitral valve repair with a transvenous, transseptal approach. In addition, several cameras, line lasers, and ultraviolet lights are used to mimic echocardiographic and fluoroscopic imaging and with a remote eye tracker the cognitive behaviour of the operator is recorded. A pilot study with a total of 9 interventional cardiologists, cardiac surgeons and technical experts was conducted. All participants performed the MitraClip procedure on the MVR simulator using standard interventional tools. Subsequently, each participant completed a structured questionnaire to assess the simulator.

**Results:**

The simulator functioned well, and the implemented objective performance assessment methods worked reliably. Key performance metrics such as x-ray usage were comparable with results from studies assessing these metrics in real interventions. Fluoroscopy imaging is realistic for the transseptal puncture but reaches its limits during the final steps of the procedure.

**Conclusion:**

The functionality and objective performance assessment of the MVR simulator were demonstrated. Especially for complex procedures such as the MitraClip procedure, this simulator offers a suitable platform for risk-free training and education.

**Supplementary Information:**

The online version contains supplementary material available at 10.1007/s13239-021-00549-4.

## Introduction

Cardiovascular disease (CVD) is the leading cause of death globally accounting for 17.9 million or 31% of all deaths in 2016.^21^ Apart from medications, physical exercise and healthier diets, many CVDs are treated surgically and thus require well-trained physicians capable of safely performing these complex interventions. Here, the potential for a healthcare crisis due to CVDs is looming as it is predicted that by 2035 the yearly cases requiring cardiovascular therapies will increase by 61% while the number of cardiovascular (CV) surgeons is expected to decrease.^12^

At the same time, technological innovations in minimally invasive surgery (MIS) are expanding indications for structural heart disease interventions, thus posing new challenges for physicians who have to master a multitude of new procedure-specific tools and complex interventional routines.^19^,^20^ In addition, during MIS the physicians are dependent upon medical imaging such as fluoroscopy or echocardiography for guidance, which requires them to be able to rapidly and efficiently process these images and master hand-eye coordination.

Because of the increased imbalance between the number of CV physicians and yearly CV surgery cases, the increasing complexity of interventional routines and other factors such as the working-hour restrictions, the traditional apprenticeship-style learning method is slowly being replaced by more competency-based methods such as simulation-based training.^1^,^20^ A simulation-based method of learning provides a safe, risk-free, and reproducible environment for aspiring physicians to improve their skills and gain valuable experience. In combination with performance assessment capabilities, such training simulators further enable an objective assessment of the physician’s performance and can provide valuable feedback.

Especially for complex interventions such as the MitraClip™ procedure (MCP), it has been shown that procedural success, procedure time and procedure complications strongly correlate with experience.^4^ However, for such complex procedures, proper simulators are missing. Currently available simulators for cardiovascular interventions can broadly be categorized into virtual, physical, or mixed simulators. Mixed simulators include both virtual and physical components. They are called ‘augmented virtual simulator’ if they are primarily virtual and they are called ‘augmented physical simulators’ if they are primarily physical.^8^ Examples for virtual and augmented virtual simulators are the Stanford Virtual Heart,^13^ the VIST^®^ Lab with the VIST^®^ G5 simulator (Mentice, Goethenburg, Sweden), the AngioMentor™ (3D Systems formerly Simbionix, Rock Hill, SC, USA), and the CathLabVR (CAEHealthcare, Montreal, Canada). Physical and augmented physical simulators include anatomical vascular models from Elastrat (Elastrat Sàrl, Genève, Switzerland), tissue models from LifeLike BioTissue (LifeLike BioTissue Inc., London, Ontario, Canada), the endovascular simulator from VivitroLabs (VivitroLabs Inc., Victoria, British Columbia, Canada), or the Heartroid^®^ from JMC Corporation (JMC Corporation, Yokohama-city, Kanagawa, Japan). However, many of these platforms do not combine the benefits from both the virtual and physical world and are not suitable for more complex interventions, such as the MCP.

Thus, we built an augmented physical simulator for the training of the entire MCP. This platform is a further development of the transseptal puncture (TSP) simulator.^22^ The desired goal of such simulators is to enable physicians to acquire the experience and skills necessary in order to perform their first real, in this case, MCP with procedural success rates, which are similar to rates from more experienced physicians. In this study, we describe the development and assessment of this simulator and its new objective performance assessment capabilities.

## Material and Methods

**Figure 1:**
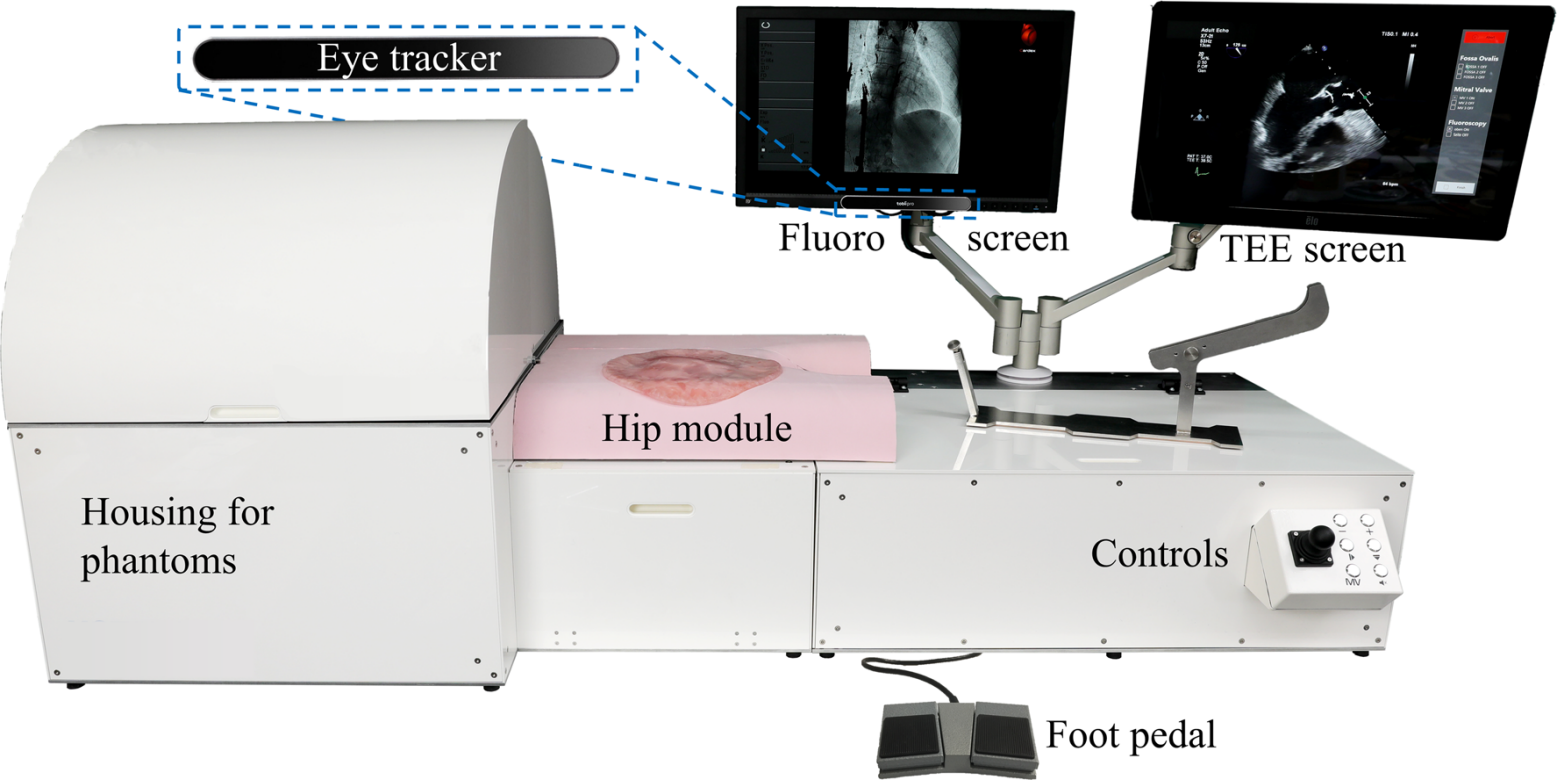
Mitral valve repair simulator. The housing containing the anatomical phantoms, cameras, line lasers and UV lights for the medical imaging simulation (details see Fig. 2). Hip module containing a femoral access catheterization pad. The screen displaying the simulated fluoroscopic images. The detailed view shows an eye tracker attached at the bottom of the screen. The screen displaying the graphical user interface and the simulated transesophageal echocardiographic images during training. Controls to switch, zoom and orient fluoroscopy view. The left foot pedal activates fluoroscopy and the right foot pedal activates a direct camera image of the anatomy.

**Figure 2:**
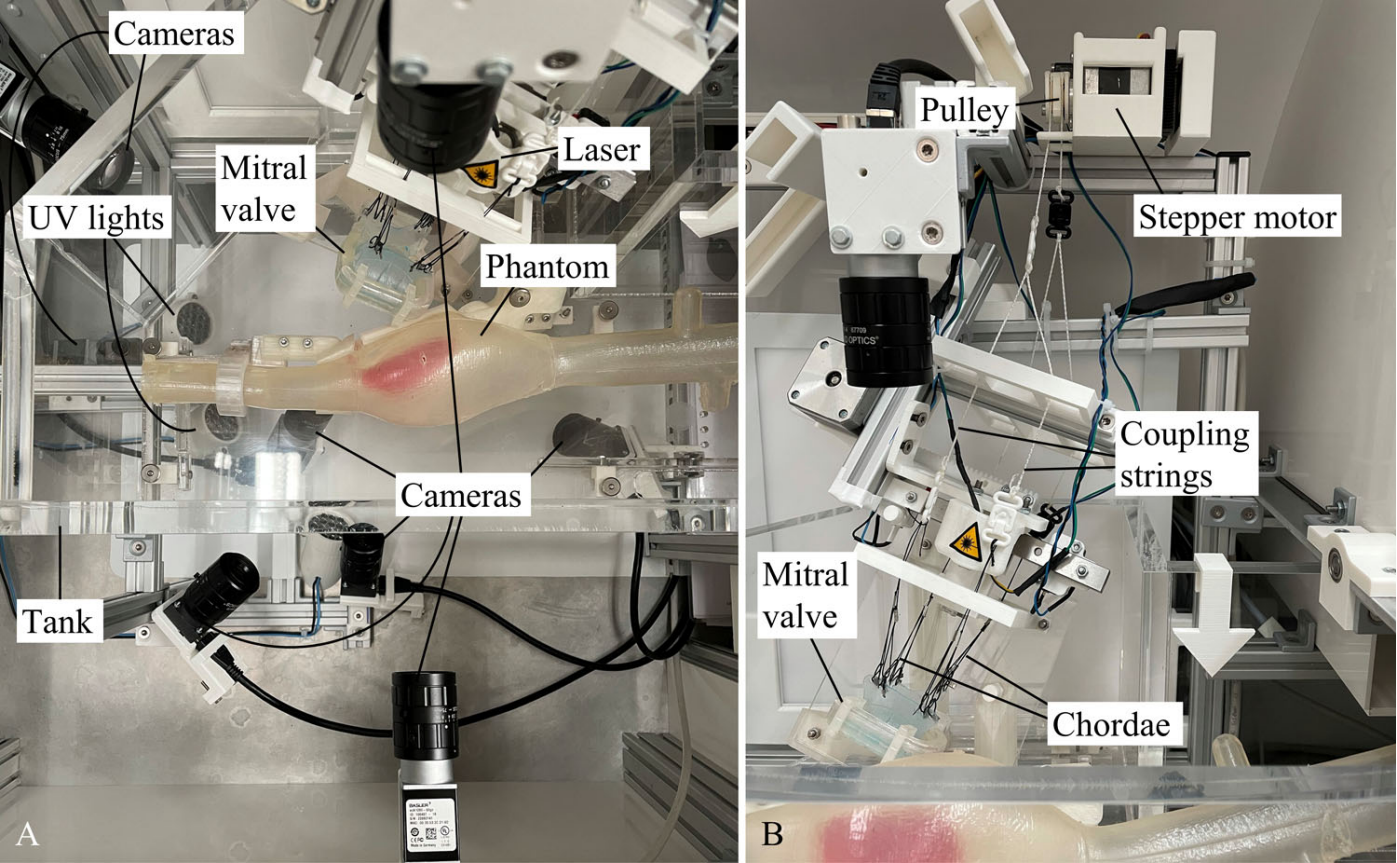
Inside views of the simulator. (a) Top view of the inside of the Mitral valve repair simulator. The simulator contains anatomical silicone phantoms of the inferior vena cava, right atrium with an exchangeable interatrial septum, superior vena cava and the mitral valve. A line laser, ultraviolet (UV) lights and 8 cameras are used to generate the necessary medical images (fluoroscopy and ultrasound) in various views for the entire intervention. (b) Front view of the inside of the Mitral valve repair simulator. Depicted are the actuation system of the mitral valve. The leaflets are connected via the chordae to a pulley by coupling springs. The pulley is actuated using a stepper motor.

The mitral valve repair (MVR) simulator is shown in Fig. 1 with its main elements highlighted. Details of the inside of the simulator are presented in Fig. 2. The hip module consists of a femoral access catheterization pad, which in turn is connected to the anatomical phantoms integrated into this simulator. Simulated fluoroscopy is displayed on the left screen and on the right screen, either the graphical user interface or the simulated transesophageal echocardiographic (TEE) images are shown to the user. The controls on the bottom right enable the operator to switch, zoom and orient the fluoroscopy views during the intervention. The fluoroscopy is activated by pressing the foot pedal.

In Fig. 2a, a top view of the insides of the simulator housing is presented. Three silicone phantoms reproduce the anatomical structures including the inferior vena cava, superior vena cava, right atrium, interatrial septum and mitral valve. In total, 8 cameras are used to simulate fluoroscopic and echocardiographic images from various viewpoints during the intervention. Additionally, ultraviolet lights and line lasers are used to highlight specific features for the imaging simulation as described in Sect. 2.2. Furthermore, during simulator use, the acrylic tank is filled until the phantoms are submerged in water in order to enhance image quality and reduce the friction between the tools and the silicone phantoms. In Fig. 2b a front view of the insides of the simulator housing is depicted. It shows the connection of the mitral valve leaflets to the actuation system, consisting of a pulley and stepper motor.

### Multi-material Anatomical Phantoms

**Figure 3:**
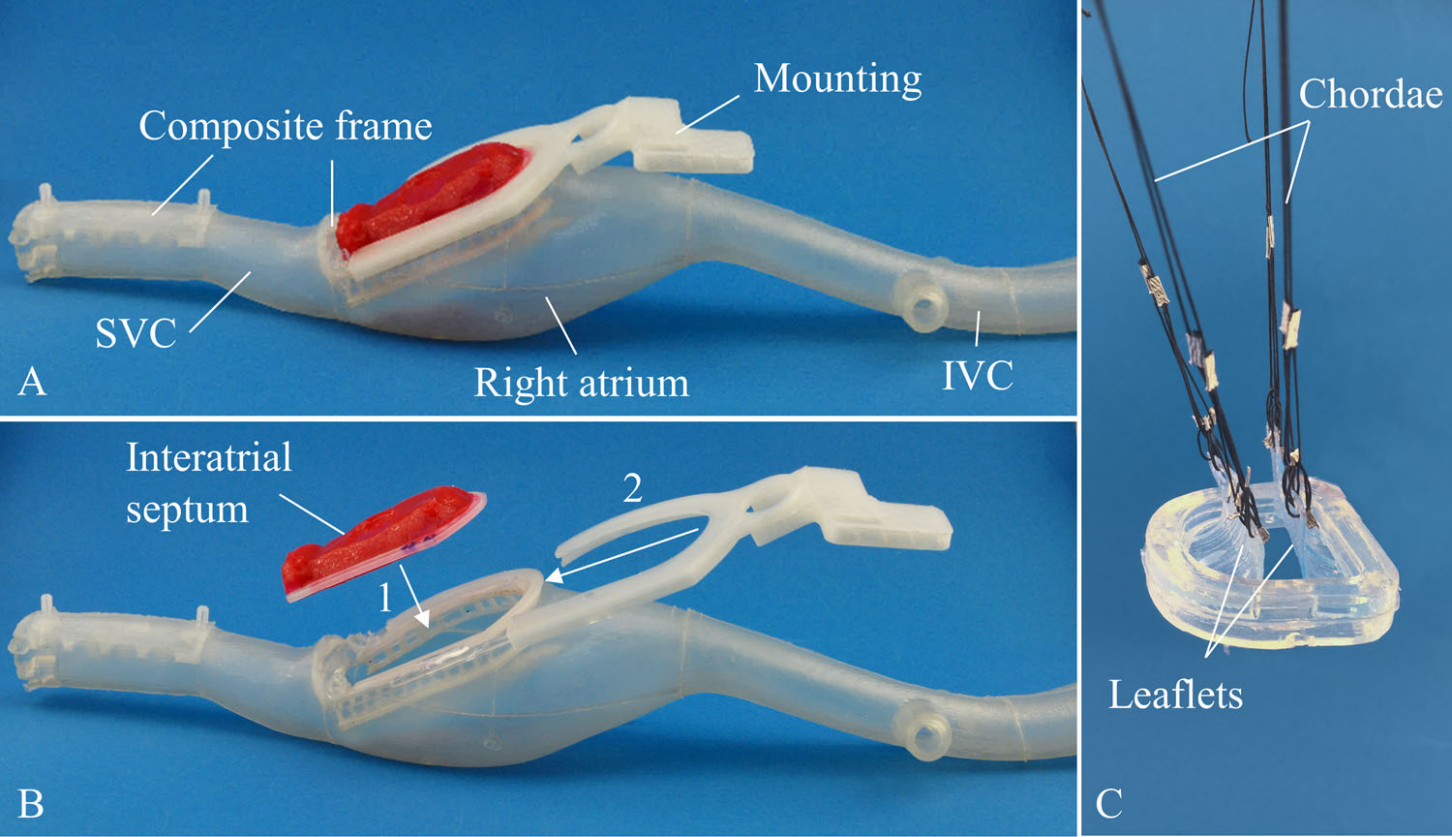
Overview of the multi-material phantoms used in the mitral valve repair simulator. (a) Silicone phantom of the inferior vena cava, right atrium, exchangeable interatrial septum, superior vena cava (SVC) and integrated rigid mountings and frames, (b) The interatrial septum can be exchanged in a two-step process. First, the mounting (2) is removed and second, the interatrial septum can be exchanged (1). (c) The mitral valve consists of a silicone annulus and leaflets which are actuated through the chordae. The chordae are realized using strings that are on one end attached to the leaflets and on the other to a stepper motor for an artificial actuation.

The anatomical structures of the MVR simulator include a hip module containing a femoral access catheterization pad, a silicone phantom representing the venous system from the femoral vein up until the superior vena cava (Fig. 3a), an exchangeable interatrial septum (Fig. 3b) and an actuated mitral valve (Fig. 3c). To derive the anatomical structures, anonymized MRI and CT data sets were obtained from the University Hospital Zurich (USZ). The data of the respective organs were segmented, fused, and converted to a 3D model. These 3D models were idolized and modified with interface connections and support structures before the final mould forms were derived and manufactured. The hip module is made of polyvinyl acetate (PVA). All the other phantoms were injection moulded and are made of silicone rubber. The phantom materials were selected based on the USZ physicians’ assessment of which material most closely resembled actual tissue. Except for the mitral valve, all phantoms include cast-in structures made of polylactide (PLA). The cast-in rigid structures provide structural stability to the right atrium and allow easy assembly and replacement of the interatrial septum. The exchangeable interatrial septum is used only once and replaced after each training procedure (illustrated in Figs. 3a–3b), as it is punctured for a successful MCP. The mitral valve is also easily replaceable and is connected via its chordae to an actuation system to simulate the movement of the valve during a cardiac cycle. The phantoms are designed as a modular system, meaning that individual parts (i.e. the interatrial septum) can easily be replaced with a new one or also a slightly different one. This enables the MVR simulator to easily be adapted to different patient scenarios. Currently, three different types of interatrial septa are available, namely with a floppy, a thin and a thick fossa ovalis. For the other phantoms, there are currently no alternatives available.

### Imaging Acquisition and Processing

In the MVR simulator, the two main imaging modalities used during transcatheter interventions, fluoroscopy and TEE, were implemented. The fluoroscopy simulation of the MVR simulator is based on the same principle as described for the TSP simulator by Zimmermann *et al.*^22^ and is therefore not described in more detail herein. The TEE simulation, however, is based on a novel technique and outlined in detail in the following section.

#### Transesophageal Echocardiography Simulation

During the MitraClip procedure, TEE is employed to help the operator guide various tools inside the patient’s body. During the TSP, TEE is crucial in determining the correct location for the puncture of the interatrial septum, while during the MitraClip placement it is needed to orient and guide the delivery device through the mitral valve and deploy the MitraClip safely. In the MVR simulator, 3 cameras in combination with ultraviolet light and line lasers generate simulated TEE images of the left ventricular outflow tract (LVOT), 0˚ and 3D view.

**Figure 4:**
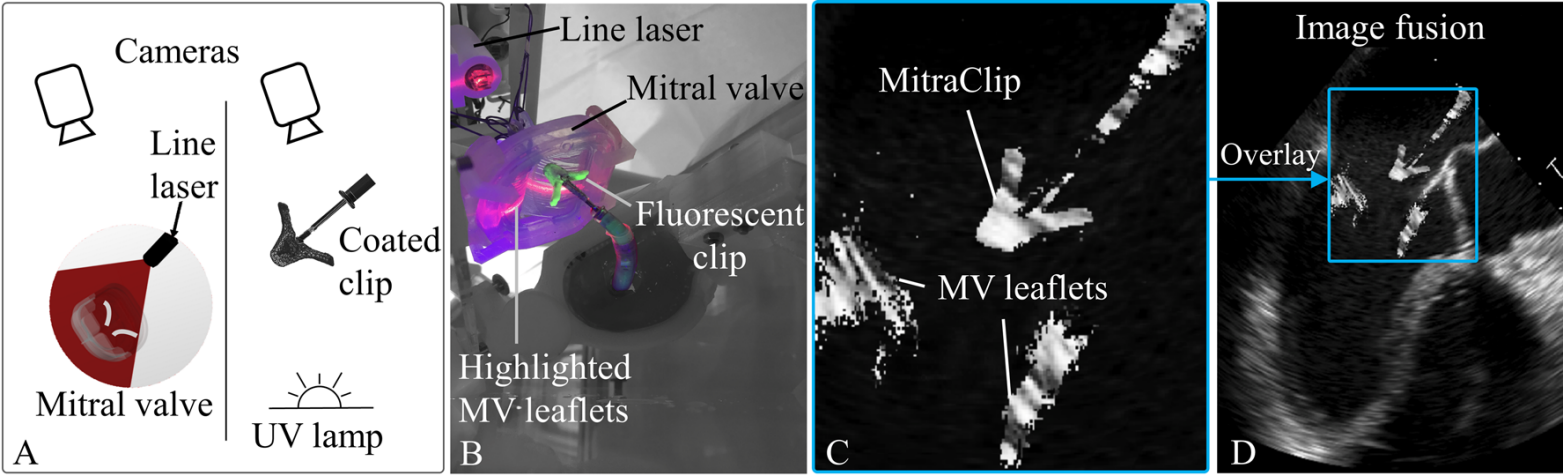
Simulated transesophageal echocardiography (TEE) workflow. (a) Illustration of the overall concept (b) Line laser creates “ultrasound like” cut through the mitral valve. Ultraviolet (UV) lights highlight the MitraClip delivery system which is coated with a fluorescent dye. (c) Recorded images of the mitral valve leaflets and the MitraClip after image colour threshold processing. (d) Overlay of simulated tools and anatomical parts shown in (c) onto a real patient TEE template to create the final simulated TEE image.

The underlying workflow for computing the TEE simulation is exemplarily illustrated in Fig. 4. The concept (Fig. 4a) is to highlight specific cross-sections of the mitral valve with a line laser (Fig 4b). In addition, the MitraClip device is coated with a fluorescent dye, which in turn is illuminated using ultraviolet lights. The highlighted cross-section and the fluorescent MitraClip device are then isolated using image processing steps to create the output shown in Fig. 4c. Finally, this output is overlaid onto a default TEE imaging template, in this case exemplarily shown for the LVOT view. The TEE imaging templates are recordings of patient TEEs for each of the implemented views. All these processing steps are computed in real-time and the resulting image is displayed to the user on the TEE screen of the MVR simulator.

### Objective Performance Assessment Capabilities

The aforementioned shift from an apprenticeship-based method of learning towards a competency-based one also means that the performance assessment is shifting from a more subjective assessment towards a more objective one. In combination with an objective structured assessment of technical skill (OSATS) or derived versions thereof, simulation-based training is making the assessment process more objective, effective and efficient.^3^,^10^

**Figure 5:**
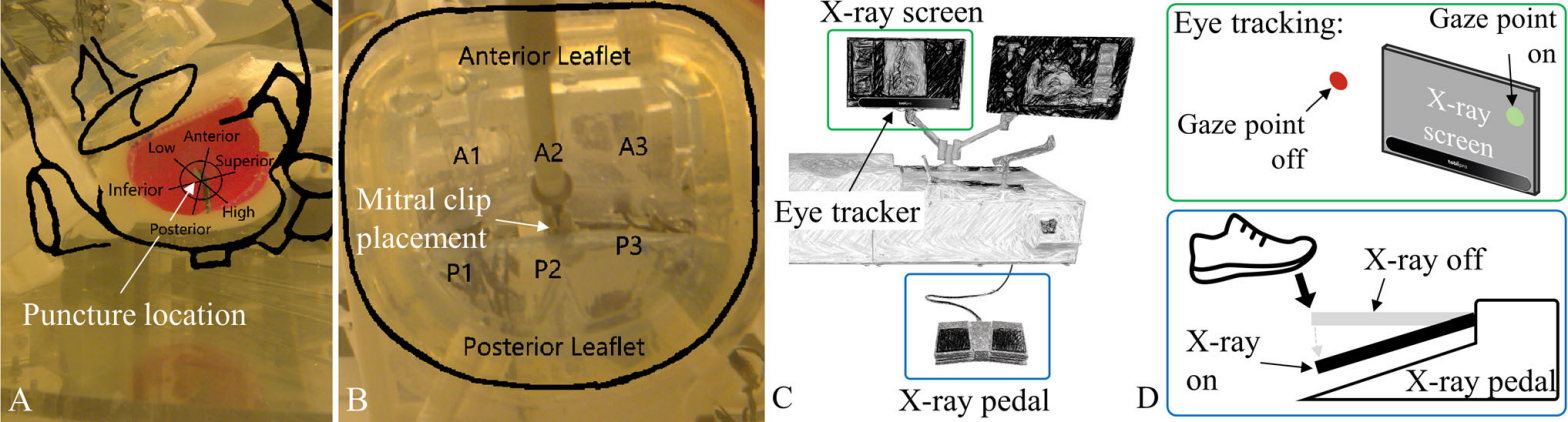
Overview of feedback capabilities of the mitral valve repair simulator. (a) Feedback image of the transseptal puncture location. The rough dimension of the left atrium, interatrial septum and mitral valve are sketched and overlaid in black. Additionally, a position grid is overlaid. (b) Feedback image of the MitraClip placement in the mitral valve. (c) Illustration of the remote eye tracker implemented on the simulator and used to compute the percentage of “correctly” used fluoroscopy. (d) Correctly used fluoroscopy means that the x-ray pedal is pressed (“X-ray on”) and at the same time the users gaze point is on the x-ray screen (“gaze point on”). If the x-ray pedal is pressed (“X-ray on”) and the gaze point is not on the x-ray screen (“gaze point off”), it is classified as unnecessary x-ray usage.

In this respect, the mitral valve repair simulator measures several metrics which can be used for an objective performance assessment. These metrics include; the total procedural time, the procedural time until the TSP, the puncture location on the interatrial septum (Fig. 5a) and the positioning of the MitraClip in the mitral valve (Fig. 5b). In addition, the simulator is equipped with a Tobii Pro Nano remote eye tracker (Tobii Technology, Danderyd Municipality, Sweden), which enables the measurement of key metrics such as the fluoroscopy time and the percentage of correctly used fluoroscopy (Fig. 5c). Correctly used fluoroscopy is defined as activating fluoroscopy and simultaneously looking at the fluoroscopy screen, meaning the physician is processing the fluoroscopy information and images (Fig. 5d).

### Pilot Study Design

The pilot study was conducted within the framework of the *Certificate of Advanced Studies–Mitral and Tricuspid Valve Structural Interventions*^16^ course at the University Hospital Zurich. The study took place in the last week of February 2020. In total 9 participants were enrolled in the pilot study and tested the MVR simulator (participant details are provided in supplementary material Table A3).

Each participant was briefed on how to use the simulator and had to sign an informed consent form before taking part in the study. Prior to the start of the test, the Tobii Pro Nano remote eye tracker was calibrated to each participant according to the calibration process described in the Tobii Pro user manual.^15^ All the tests were performed with the same medical tools used in a real MCP. This included; a 150 cm of 0.9 mm (0.035″) GuideRightTM J-tip guidewires (Abbott Laboratories, Chicago, IL), a Swartz TM braided transseptal guiding introducer (Abbott Laboratories, Chicago, IL), a BRK-1TM transseptal needle, a 260 cm of 0.9 mm (0.035”) super-stiff exchange length guidewire, and a MitraClip system (Abbott Vascular, Santa Rosa, CA) consisting of a steerable guide catheter and a clip delivery system. The MVR simulator was equipped with the same silicone phantoms and catheterization pad for all procedures; however, the interatrial septum was exchanged for a new one after each MCP. The MCP was finished when the clip was implanted on the mitral valve and the clip position was deemed satisfactory by the participant. The procedure did not include the clip deployment, as this step is irreversible and would have required a new clip delivery system for each procedure. A movie showing excerpts of an entire MCP performed on the MVR simulator is provided as supplementary material *“MitralValveRepairSimulator-Procedure.mp4”*.

After the experiment, each participant filled out an anonymized, structured questionnaire consisting of 27 items on a 7-point Likert scale and 5 open questions. The 27 items were assigned to either the face or content validity category for the subsequent analysis (the full questionnaire is provided as supplementary material *“Structured_questionnaire.pdf”*). All items were weighted equally, and a value of 4.0 was defined as a minimum so that a category can be regarded as sufficient for the simulator. The questionnaire was adapted to cover MCP and is based on typical structures for face, content and criterion validation study in the field of medical simulators.^6^,^7^,^11^,^17^

## Results

Scores given to the MVR simulator are summarized in Table 1, listed for the different categories. Detailed scores for each question of the structured questionnaire are provided as supplementary material Table A4. Overall, the MVR simulator received high scores of between 5.25 and 5.5 (out of 7) in both validation categories.

**Table 1:** Validation category-specific results from the structured questionnaire

Validity	Mean	Standard deviation	Minimum	Maximum
Face	5.28	0.76	3.63	6.55
Content	5.48	0.74	4.25	6.63

Scores were given on a 7-point Likert scale

**Figure 6:**
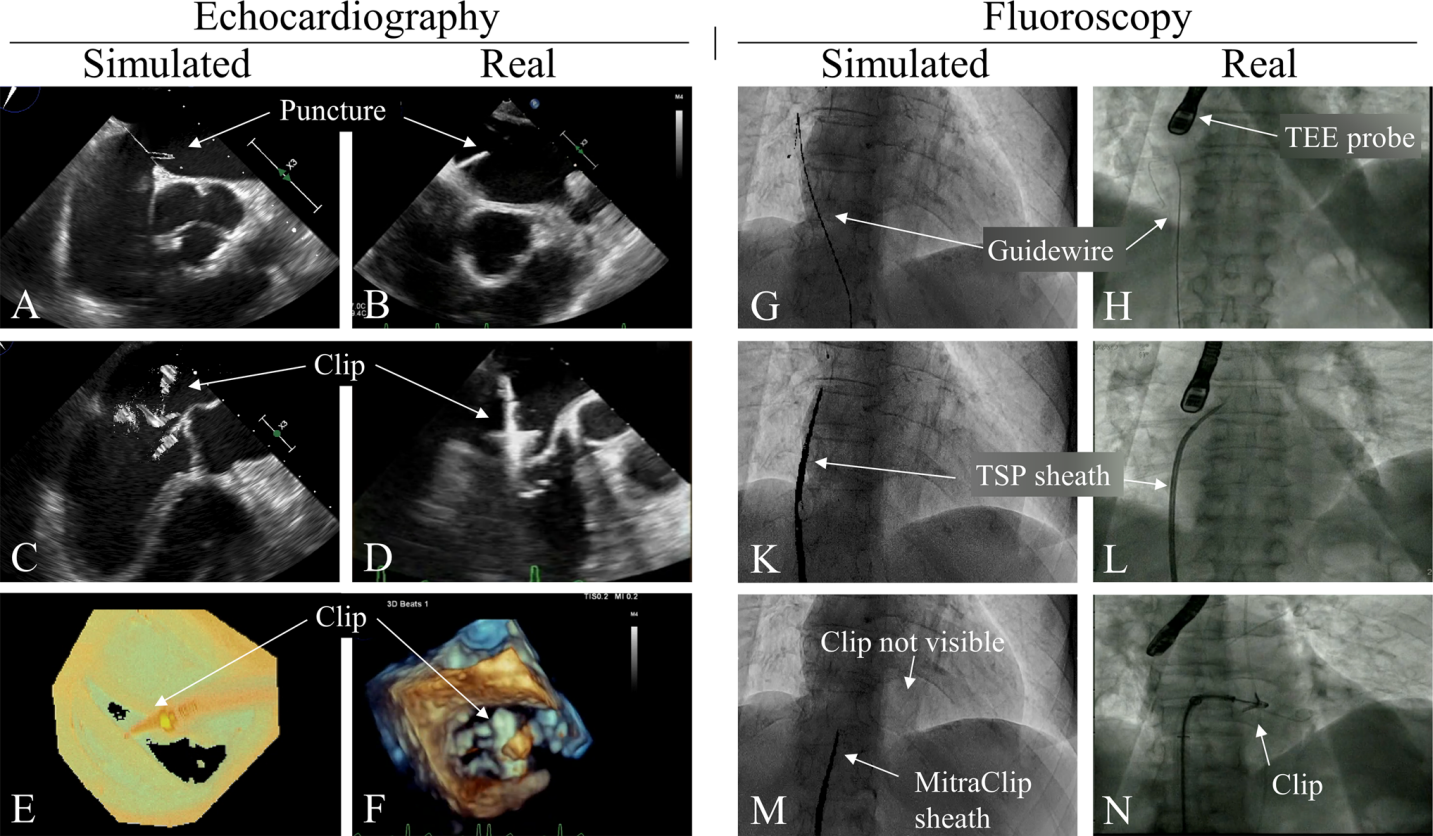
Comparison between the MVR simulator simulated and real echocardiography (echo) and fluoroscopy (fluoro) images. (a–b) Simulated and real echo image of the transseptal puncture. (c–d) Simulated and real echo image of the MitraClip right above the mitral valve. (e–f) Simulated and real 3D echo view looking down onto the mitral valve. (g–h) Simulated and real fluoro image of a guidewire inside the right atrium. (k–l) Simulated and real fluoro image of the TSP sheath right before puncture. (m–n) Simulated and real image of the MitraClip inside the left atrium and ventricle. The simulation fails to emulate the clip inside the left atrium.

One consistently lower-rated aspect of the MVR simulator is the imaging simulation. Specifically, the fluoroscopy simulation was oftentimes scored as unsatisfactory. In Fig. 6, simulated echocardiography and fluoroscopy images are shown next to real images from various procedural steps. The simulated images look very similar compared to the real ones. However, for the echocardiography images there is sometimes a mismatch in terms of texture and colouring (Figs. 6a–6b: thinner puncture lines, Figs. 6c–6d: difference in clip texture, Figs. 6e–6f: difference in colouring). The fluoroscopy images are very similar to the real ones during guidewire insertion and TSP sheath advancement (Figs. 6g–h and 6k–l). However, the current system is not able to accurately simulate the fluoroscopy images once the MitraClip is advanced into the left atrium and ventricle (Figs. 6m–6n: clip is not visible). Additionally, a video comparing simulated to real fluoroscopy and echocardiography is provided as supplementary material *“MitralValveRepairSimulator-Imaging.mp4”*.

As described in Sect. 2.3 the MVR simulator measured fluoroscopy usage data using a remote eye tracker. The derived results from this objective performance assessment tool are listed in Table 2.

**Table 2:** Fluoroscopy usage times of participants

Metric	Results (mean ± SD)
X-ray “On” time (s)	560 ± 187
X-ray “used” time (s)	300 ± 66
Correct X-ray actuation (%)	58 ± 10

X-ray “On” time is the total time the fluoroscopy simulation was on (meaning the foot pedal was pressed). X-ray “used” time indicates the actual time where fluoroscopy was on and the operator looked at the fluoroscopy screen. Correct X-ray actuation is the percentage of “correctly” used fluoroscopy

In summary, the use of fluoroscopy is far from ideal as the average of correctly activated x-rays is slightly above half with 58%.

In addition to the 7-point Likert scale statements of the questionnaire, the participants also answered several open questions. The results from these are summarized qualitatively. The main points of concerns or wishes for improvements were related to the imaging simulation, the MitraClip system and the simulation immersion. Specifically, the used MitraClip system was deteriorating due to the extensive use, this resulted in the occasional jamming of the clip. In terms of simulation immersion, several participants indicated that the training scenario could benefit from including case-specific information and storyline.

## Discussion

The presented platform is the first physical augmented simulator for complex mitral valve repair interventions, such as the MitraClip procedure. The functionality and capabilities of this simulator were demonstrated in a pilot study. The study participants rated various aspects related to face and content validity and its scores, 5.28 ± 0.76 (out of 7) and 5.48 ± 0.74 respectively, compare well with validation scores of other medical training simulators.^2^,^5^,^18^,^22^ However, as these results are based solely on the pilot study this first must be demonstrated in a larger study to more strongly support this comparison. Furthermore, it became apparent that the capabilities of the current method of simulating the fluoroscopy images need improvements. The results indicate that for more complex intervention routines, this method is not capable of creating realistic enough images and thus, one might need to consider other ways of simulating fluoroscopy in these settings. For less complex interventional routines, such as the TSP, this method works well. Moreover, the novel method for simulating the echocardiography can create realistic images even for complex interventional routines.

In terms of objective performance assessment, the eye tracking based assessment metric worked reliably and delivered valuable, additional feedback to the users. Results from the pilot study show that only 58% of the time the fluoroscopy system was used correctly. This means that around 40% of the time when the x-ray was active, the participants did not look at the fluoroscopy screen and therefore weren’t able to process the information. These findings compare well with what other studies observed in real settings, in that up to one-third of all CT scans are unnecessary and are performed without good medical justification^9^,^14^ and that 43.5% of the x-ray usage time in fluoroscopy guided cardiovascular interventions is in fact avoidable.^23^ This indicates that the eye tracking system works and is potentially able to sensitize and train physicians to mitigate unnecessary radiation usage during fluoroscopy guided procedures.

With this presented pilot study we demonstrate the operability and functional capabilities of an MVR simulator. The pilot study results indicate good face and content validity. However, in the next step the face, content and construct validity need to be assessed in a more comprehensive study that involves a control group to determine the outcomes of simulation-based training on the simulator compared to conventional training approaches. With the aim to prove that training with the MVR simulator correlates to improved physician performance and procedural outcome.

Moreover, it should be noted that with respect to the 3R principles (i.e. reduce, refine, replace) for the use of animals for intervention training, simulators play a crucial role, but they cannot currently replace them entirely. Performing an intervention on animals still offers a higher-fidelity experience than performing one on a simulator. However, with the advent of higher-fidelity simulators, the use of animals for intervention training may be reduced and, in the long run, may be replaced altogether.

**Study Limitations** Limitations of the MVR simulator itself include inherent limitations regarding the fluoroscopy imaging simulation, and no true physiological metrics, such as pressure readings or heart rate, are measured yet and displayed to the physician during the intervention.

## Conclusion

We presented an augmented physical simulator, based on anatomical phantoms and simulated imaging modalities, for complex MVR interventions such as the MCP. The simulator is equipped with new eye tracking based objective performance assessment tools. In a pilot study, a total of 9 participants trained on the MVR simulator and subsequently assessed its capabilities. Overall, the participants reported good scores in the face and content validation categories. With the eye tracking based assessment tool, similar results were observed as in the real operating room, indicating that it could be an important tool in training prospective physicians to mitigate unnecessary radiation during fluoroscopy guided interventions. Collectively, these results indicate that the MVR simulator may be a suitable tool for simulation-based training of complex catheter-based cardiovascular interventions, however, further validation studies are needed to substantiate this.

## Supplementary Information

Below is the link to the electronic supplementary material.

Supplementary material 1 (DOCX 35 kb)

Supplementary material 2 (PDF 766 kb)

Supplementary material 3 (MP4 63474 kb)

Supplementary material 4 (MP4 133793 kb)

## Abbreviations

CV: Cardiovascular
CVD: Cardiovascular disease
LVOT: Left ventricular outflow tract
MIS: Minimally invasive surgery
MCP: MitraClip procedure
MVR: Mitral valve repair
OSATS: Objective structured assessment of technical skill
PLA: Polylactide
PVA: Polyvinyl acetate
TSP: Transseptal puncture
TEE: Transesophageal echocardiography
USZ: University Hospital Zurich
