# Supplementary material for: Transcatheter Mitral Valve Repair Simulator Equipped with Eye Tracking Based Performance Assessment Capabilities: A Pilot Study
Source: Cardiovasc Eng Technol. 2021 Jun 7;12(5):530–8. doi: 10.1007/s13239-021-00549-4 (PMC8481152; doi:10.1007/s13239-021-00549-4)
Supplement: Supplementary file 1 — Supplementary material 1 (DOCX 35 kb) [file 13239_2021_549_MOESM1_ESM.docx]

## Appendices

### Tables

Table A3 Detailed list of the participants of the pilot study. This includes the years of experience and the number of transseptal and the number of MitraClip interventions performed.

| **Participant** | **Profession** | **Experience (years)** | **Number of TSP** | **Number of MCI** |
| --- | --- | --- | --- | --- |
| P01 | Int. cardiologist | 12 | 80 | 20 |
| P02 | Int. cardiologist | 5 | 70 | 90 |
| P03 | MC representative | N.A. | 0 | 0 |
| P04 | Int. cardiologist | 5 | 500 | 150 |
| P05 | Int. cardiologist | 10 | 50 | 5 |
| P06 | MC representative | 28 | 100 (animals) | 1000 (animals) |
| P07 | MC representative | 13 | 10 (animals) | 0 |
| P08 | Cardiac surgeon | 30 | 1000 | 800 |
| P09 | Cardiac surgeon | 2 | 10 | 1 |
| *TSP: transseptal puncture, MCI: MitraClip intervention, Int.: interventional, MC: MitraClip* | | | | |

Table A4 Detailed scores of all the participants' responses to the 7-Point Likert scale items of the structured questionnaire of the pilot study. The listed question number corresponds to the numbering in the structured questionnaire (provided as supplementary material “Structured_questionnaire.pdf”). The category depicts to which validity category the respective question was assigned to. Questions 38-41 are related to the criterion validity, however, because of too few data points, they were excluded in the analysis of this study.

| **Question Nr.** | **Category** | **P01** | **P02** | **P03** | **P04** | **P05** | **P06** | **P07** | **P08** | **P09** |
| --- | --- | --- | --- | --- | --- | --- | --- | --- | --- | --- |
| 11 | Face | 5 | 7 | 6 | 5 | 4 | 4 | 5 | 6 | 5 |
| 12 | Face | 6 | 6 |  | 6 | 5 | 7 | 6 | 7 | 5 |
| 13 | Face | 6 | 6 | 6 | 5 | 4 | 2 | 5 | 6 | 4 |
| 14 | Face | 5 | 7 | 7 | 5 | 4 | 7 | 5 | 7 | 5 |
| 15 | Content | 4 | 5 | - | 6 | 3 | 2 | 5 | 6 | 4 |
| 16 | Content | 7 | 6 | 6 | 7 | 7 | 5 | 6 | 7 | 6 |
| 17 | Face | 6 |  |  | 3 | 4 | - | 6 | 6 | 5 |
| 18 | Face | 4 | 3 | - | 6 | 4 | 4 | 5 | 7 | 4 |
| 19 | Content | 5 | 6 | - | 5 | 5 | 6 | 6 | 5 | 5 |
| 20 | Content | 6 |  | - | 4 | 5 | 6 | 6 | 5 | 4 |
| 22 | Face | 2 | 5 | - | 2 | 5 | 2 | 5 | 4 | 4 |
| 23 | Content | 5 | 4 | - | 1 | 5 | 2 | 6 | 5 | 6 |
| 24 | Face | 7 | 7 | 6 | 4 | 3 | 3 | 6 | 5 | 4 |
| 25 | Content | 4 | 7 | 5 | 5 | 4 | 5 | 6 | 6 | 6 |
| 27 | Face | 6 | 7 | 6 | 6 | 5 | 6 | 6 | 6 | 5 |
| 28 | Content | 7 | 7 | 6 | 7 | 5 | - | 6 | 6 | 5 |
| 29 | Content | 7 | 7 | 6 | 7 | 5 | - | 6 | 6 | 7 |
| 31 | Face | 7 | 7 | 6 | 7 | 5 | 6 | 7 | 7 | 7 |
| 32 | Face | 7 | 6 | 4 | 6 | 3 | 5 | 6 | 7 | 5 |
| 33 | Content | 6 | - | - | 5 | 5 | 2 | 6 | 5 | 5 |
| 34 | Content | 6 | 6 | - | 6 | 5 | 3 | 6 | 6 | 4 |
| 35 | Content | 7 | 7 | - | 7 | 7 | 6 | 6 | 7 | 6 |
| 36 | Content | 7 | 5 | - | 5 | 6 | 5 | 6 | 7 | 5 |
| 38 | Criterion | 6 | - | - | - | - | - | - | 6 | 5 |
| 39 | Criterion | 6 | - | - | - | - | - | - | 7 | 5 |
| 40 | Criterion | 7 | - | - | - | - | - | - | 7 | 5 |
| 41 | Criterion | 6 | - | - | - | - | - | - | 6 | 5 |
