# Supplementary material for: Transcatheter Mitral Valve Repair Simulator Equipped with Eye Tracking Based Performance Assessment Capabilities: A Pilot Study
Source: Cardiovasc Eng Technol. 2021 Jun 7;12(5):530–8. doi: 10.1007/s13239-021-00549-4 (PMC8481152; doi:10.1007/s13239-021-00549-4)
Supplement: Supplementary file 2 — Supplementary material 2 (PDF 766 kb) [file 13239_2021_549_MOESM2_ESM.pdf]

Mitra valve repair simulator questionnaire

Please read each statement carefully and indicate your level of agreement or disagreement by circling the appropriate number on the scale below.

*Required

General information

1. Simulation Date * - (Filled in by supervisor)

   Example: 7 January 2019

2. Participant Nr. * - (Filled in by supervisor)

3. Fluoroscopy Time (s) - (Filled in by supervisor)

4. Correctly used Fluoroscopy Time (s) - (Filled in by supervisor)

5. Percentage of correctly used fluoroscopy - (Filled in by supervisor)

6. Profession *

   Mark only one oval.

   - Cardiac Surgeon
   - Interventional Cardiologist
   - Non-Interventional Cardiologist
   - Other: ______________

7. How many years in practice?

8. How many Transseptal Punctures(TSP)s have you performed in your career? *

9. How many MitraClip Implantations (MCI) have you performed in your career? *

Overall Impression

10. In a few words, what is your overall impression of the simulator? (2 positive aspects, 2 improvable aspects)

11. The simulator setting is a realistic representation of the workplace

Mark only one oval.

	1	2	3	4	5	6	7	
Strongly Disagree	◯	◯	◯	◯	◯	◯	◯	Strongly Agree

12. I had to perform the same procedural steps as in the OR

Mark only one oval.

	1	2	3	4	5	6	7	
Strongly Disagree	◯	◯	◯	◯	◯	◯	◯	Strongly Agree

13. Performing MCI on the simulator is a realistic representation of the procedure

Mark only one oval.

	1	2	3	4	5	6	7	
Strongly Disagree	◯	◯	◯	◯	◯	◯	◯	Strongly Agree

Tools and Haptics

14. The procedural tools used during the simulation felt realistic

Mark only one oval.

	1	2	3	4	5	6	7	
Strongly Disagree	◯	◯	◯	◯	◯	◯	◯	Strongly Agree

15. The simulation allowed me to adequately demonstrate my technical skills

Mark only one oval.

	1	2	3	4	5	6	7	
Strongly Disagree	◯	◯	◯	◯	◯	◯	◯	Strongly Agree

16. The simulator is a good device to train technical skills

Mark only one oval.

	1	2	3	4	5	6	7	
Strongly Disagree	◯	◯	◯	◯	◯	◯	◯	Strongly Agree

17. The haptic feedback during the TSP procedure was realistic

Mark only one oval.

	1	2	3	4	5	6	7	
Strongly Disagree	◯	◯	◯	◯	◯	◯	◯	Strongly Agree

18. The haptic feedback during the MCI procedure was realistic

Mark only one oval.

	1	2	3	4	5	6	7	
Strongly Disagree	⬭	⬭	⬭	⬭	⬭	⬭	⬭	Strongly Agree

Anatomy

19. The Anatomical Structure up to the moment of TSP felt right

Mark only one oval.

	1	2	3	4	5	6	7	
Strongly Disagree	⬭	⬭	⬭	⬭	⬭	⬭	⬭	Strongly Agree

20. The Anatomical Structure between the moment of TSP and Clip placement felt right

Mark only one oval.

	1	2	3	4	5	6	7	
Strongly Disagree	⬭	⬭	⬭	⬭	⬭	⬭	⬭	Strongly Agree

21. One point you would improve in the anatomy

____

Imaging

22. The fluoroscopic imaging was realistic

Mark only one oval.

	1	2	3	4	5	6	7	
Strongly Disagree	⬭	⬭	⬭	⬭	⬭	⬭	⬭	Strongly Agree

23. The provided fluoroscopic images are useful

Mark only one oval.

	1	2	3	4	5	6	7	
Strongly Disagree	⬭	⬭	⬭	⬭	⬭	⬭	⬭	Strongly Agree

24. The transesophageal echocardiography (TEE) imaging was realistic

Mark only one oval.

	1	2	3	4	5	6	7	
Strongly Disagree	⬭	⬭	⬭	⬭	⬭	⬭	⬭	Strongly Agree

25. The provided TEE images are useful

Mark only one oval.

	1	2	3	4	5	6	7	
Strongly Disagree								Strongly Agree

26. One point you would improve in the imaging

____

User Interface

27. The User Interface is intuitive

Mark only one oval.

	1	2	3	4	5	6	7	
Strongly Disagree								Strongly Agree

28. The Feedback gives valuable information to the direct user

Mark only one oval.

	1	2	3	4	5	6	7	
Strongly Disagree								Strongly Agree

29. The Feedback gives valuable information to a trainee supervisor

Mark only one oval.

	1	2	3	4	5	6	7	
Strongly Disagree								Strongly Agree

30. One point you would improve in the feedback to the user

____

Training aspect

31. The simulator is good for training novice physicians

Mark only one oval.

	1	2	3	4	5	6	7	
Strongly Disagree								Strongly Agree

32. The simulator is good for training expert physicians

Mark only one oval.

	1	2	3	4	5	6	7	
Strongly Disagree								Strongly Agree

33. The simulation is an accurate judge of my overall competences in performing TSP

Mark only one oval.

	1	2	3	4	5	6	7	
Strongly Disagree	⬭	⬭	⬭	⬭	⬭	⬭	⬭	Strongly Agree

34. The simulation is an accurate judge of my overall competence in performing MCI

Mark only one oval.

	1	2	3	4	5	6	7	
Strongly Disagree	⬭	⬭	⬭	⬭	⬭	⬭	⬭	Strongly Agree

35. Do you feel that the simulator is useful in teaching the correct manipulation of procedural tools

Mark only one oval.

	1	2	3	4	5	6	7	
Strongly Disagree	⬭	⬭	⬭	⬭	⬭	⬭	⬭	Strongly Agree

36. Do you feel that the simulator is useful in teaching hand-eye coordination

Mark only one oval.

	1	2	3	4	5	6	7	
Strongly Disagree	⬭	⬭	⬭	⬭	⬭	⬭	⬭	Strongly Agree

Compared to other simulators

37. Do you have any experience with other TSP or Mitraclip simulators?

Mark only one oval.

⬭ Yes
⬭ No

38. If yes: how would you rate this simulator's imaging compared to the ones you have already worked on?

Mark only one oval.

	1	2	3	4	5	6	7	
Terrible	⬭	⬭	⬭	⬭	⬭	⬭	⬭	Excellent

39. If yes: how would you rate this simulator's haptic feedback compared to the ones you have already worked on?

Mark only one oval.

	1	2	3	4	5	6	7	
Terrible	⬭	⬭	⬭	⬭	⬭	⬭	⬭	Excellent

40. If yes: how would you rate this simulator's technical steps compared to the ones you have already worked on?

Mark only one oval.

	1	2	3	4	5	6	7	
Terrible	⬭	⬭	⬭	⬭	⬭	⬭	⬭	Excellent

41. If yes: how would you rate this simulator compared to the ones you have already worked on?

Mark only one oval.

	1	2	3	4	5	6	7	
Terrible	⬭	⬭	⬭	⬭	⬭	⬭	⬭	Excellent

42. What would you wish this simulator had compared to others?

____
